# Surface electromyographic evaluation of jaw muscles in children with unilateral crossbite and lateral shift in the early mixed dentition. Sexual dimorphism

**DOI:** 10.4317/medoral.17942

**Published:** 2012-08-28

**Authors:** Leticia Lenguas, José-Antonio Alarcón, Filipa Venancio, Marta Kassem, Conchita Martín

**Affiliations:** 1 Department of Stomatology IV, School of Dentistry, University Complutense of Madrid, Madrid, Spain; 2 Department of Stomatology, Area of Orthodontics, School of Dentistry, University of Granada, Granada, Spain

## Abstract

Objectives: To examine the activity of jaw muscles at rest and during maximal voluntary clenching (MVC) in children with unilateral posterior crossbite (UPXB) and functional lateral shift in the early mixed dentition and to evaluate sex differences. Material and Methods: The sample included 30 children (15 males, 15 females) aged 6 to 10 years old, with UPXB and functional mandibular lateral shift (≥1.5 mm) in the early mixed dentition. sEMG activity coming from the muscle areas (anterior temporalis [AT], posterior temporalis [PT], masseter [MA] and suprahyoid [SH]) were obtained from both the crossbite (XB) and noncrossbite (NONXB) sides at mandibular rest position. sEMG acti-vity of the bilateral AT and MA muscles sides was obtained during MVC. Asymmetry and activity indexes were calculated for each muscle area at rest and during MVC; the MA/TA ratio during MVC was also determined. Results: At rest, no differences were found between sexes for any muscle areas or asymmetry and activity indexes. No differences were found between XB and NONXB sides. During MVC, however, significant sex differences were found in AT and MA activity, with higher sEMG values in males than in females, on both XB and NONXB sides. Asymmetry indexes, activity indexes and MA/AT ratios did not show significant differences between the sexes. Activity was symmetric both in males and in females. Conclusions: At rest, no sex differences were found, but during MVC males showed higher activity than did females in both XB and NONXB AT and MA muscle areas. Muscular activity was symmetrical at rest and during MVC in both sexes. Sexual dimorphism should be considered in the diagnosis and treatment of UPXB and lateral shift in the early mixed dentition.

** Key words:**Unilateral crossbite, mandibular shift, jaw muscles, sEMG, early mixed dentition.

## Introduction

Unilateral posterior crossbite (UPXB) is a frequent malocclusion in the early mixed dentition stage (1,2) that normally persists from childhood to adulthood if not treated, although some authors have found instances of spontaneous correction after eliminating dummy (pacifier)- and finger-sucking (3). In most cases it is accompanied by a functional mandibular lateral shift during closure toward the crossbite side (4) that has been associated with certain side effects such as asymmetrical mandibular growth (5,6), asymmetrical jaw muscle activity (7-10), changes in mandibular rest position and movements (11) and temporomandibular joint (TMJ) disorders (12). In some studies the anterior temporalis (AT) thickness was lower on the crossbite (XB) side than on the non crossbite (NONXB) side at rest (13), while the masseter (MA) muscle was thinner on the XB side than on the NONXB side during maximal clenching (14). A reduction in bite force was also described in UPXB children in the mixed dentition (15).

Factors such as age, type of dentition and gender can influence development of the jaw muscles. It is well known that adult males have a larger bite force than do females (16,17), probably due to thicker and more abundant type II fibers in the males’ MA muscles (18). These differences in adult MA fiber profile may be attributed to genetic factors (19) and sex hormones (18). Other authors have reported differences in muscle length and bulk between adult males and females that would explain these differences during isometric bites (20,21). Nevertheless, in a group of 7- to 12-year-old children Palinkas et al. (17) did not find any sex differences in bite force.

Some sEMG studies of healthy, normo-occlusive adults have found a higher MA activity in males compared with females during clenching (22-24); others found a higher activity of both MA and AT in males than in females (25); still others found no significant differences according to sex (16).

At rest position, no sex differences have been reported in sEMG activity of AT and MA in normo-occlusive adults (22,23,26).

Despite the potential side effects of an untreated UPXB associated with functional lateral shift, few neuromuscular studies have been carried out in children with UPXB in the early stages of their occlusal development. Furthermore, despite the possible influence of gender in jaw muscle development, we found no such studies in children with UPXB.

Therefore, the purposes of this study were to examine sEMG activity of jaw muscles at rest and during maximal voluntary clenching (MVC) in children with UPXB and functional mandibular lateral shift in the early mixed dentition and to evaluate sex differences.

## Material and Methods

-Patient population

Thirty Caucasian children (15 males, 15 females) aged 6 to 10 years in the early mixed dentition, diagnosed with UPXB and functional mandibular lateral shift (≥1.5 mm) to the XB side were recruited from referrals to the Pediatric Clinic at the School of Dentistry of the University Complutense of Madrid. Posterior XB was diagnosed by the identification of at least one posterior tooth (from primary canine to permanent first molar) in full XB, that is, the buccal cusp of the maxillary tooth occluding lingually to the buccal cusp of the corresponding mandibular tooth.

Exclusion criteria were the presence of skeletal asymmetries (measured on frontal and Hirtz radiographs), craniofacial anomalies, TMJ dysfunction, history of neuromuscular disease or disease affecting neuromuscular performance, dental caries, extensive restorations, dental pain, previous or current orthodontic treatment and deciduous tooth mobility during functional evaluation.

Parents signed their informed consent to the participation of their children in the study, which was approved by the ethics committee of University Complutense.

-Mandibular shift evaluation

Mandibular lateral shift was tested using a Kinesiograph computer system (K6-I Diagnostic System, Myotronics-Noromed, Kent, WA), according to a previously described protocol (27). Functional mandibular lateral shift was defined as the difference (mm) between lateral shift of the mandible from maximum opening to maximal intercuspation. One calibrated examiner (M.C.M.) performed all Kinesiographic measurements in a ‘‘blind’’ manner, i.e., unaware of the presence of a posterior XB. Reproducibility of the Kinesiographic records was tested by comparing the results of two consecutive measurements of 10 randomly selected subjects.

-Electromyography study

The study was performed with an EM2 electromyograph (K6-I Diagnostic System, Myotronics-Noromed, Kent, WA), with eight channels and a frequency bandwidth response of 45–430 Hz per channel that allows four pairs of muscles to be tested simultaneously. Disposable, 10-mm-diameter silver/silver chloride bipolar surface electrodes (Duo-Trode, Myotronics-Noromed) were positioned (interelectrode distance, 21 ± 1 mm) on the muscle bellies parallel to the muscle fibers according to a previously described protocol (9,27). Simultaneous surface electromyographic activity coming from the XB and NONXB sides’ AT, posterior temporalis (PT), MA and suprahyoid (SH) muscle areas were obtained at mandibular rest position. Then, sEMG activity from the bilateral AT and MA was obtained during MVC in maximal intercuspation. Asymmetry and activity indexes (28) were calculated for each muscle area at rest and during MVC; the MA/TA ratio during MVC was also determined. One calibrated examiner (M.C.M.) performed all EMG measurements without knowing of the presence or absence of UPXB, and patient data remained blinded throughout the analysis. To test the reproducibility of sEMG data, five subjects underwent four trials over 4 days following the experimental protocol.

-Statistical analysis

SPSS 19.0 software (SPSS Inc, Chicago, IL) was used for statistical analysis. Means and 95% confidence intervals (CIs) were calculated for resting and MVC sEMG values. After establishing the normal distribution of variables by means of the Shapiro-Wilks test, data were compared between groups (boys and girls) and between sides in each group by using a t test for independent samples, a paired t test, or Mann Whitney and Wilcoxon tests. The paired t test was used to assess kinesiographic measurement reproducibility and ANOVA tests for repeated measurements. Then the Tukey-Kramer multiple-comparison test was used to test sEMG measurement reproducibility. Significance was set at the 5% level (p ≤ .05).

## Results

Statistical analysis showed no significant differences among repeated sEMG or kinesiographic recordings.

-Mandibular shift

No significant differences were found in the amount of mandibular lateral shift, ranging from 1.95 to 3.24 mm (median, 2.59 mm) in males and from 2.12 to 4.60 mm (median, 3.36 mm) in females ( Table 1).

**Table 1:**
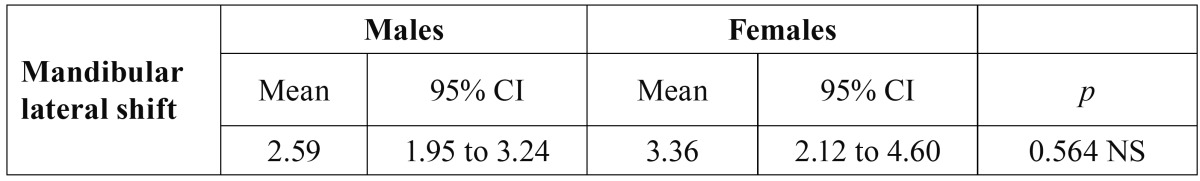
Comparisons of mandibular lateral shift (mm) between sexes.

-Rest position

 Table 2 shows the mean values and 95% CI of sEMG recorded in the eight examined muscle areas at rest. When comparing males and females, no significant differences were found in any of the tested muscle areas. A predominance of PT activity could be seen in both sexes. As no differences by sex were found, we compared the two gender groups by sEMG differences between XB and NONXB sides, and no differences were found in the muscle areas ( Table 3).

**Table 2:**
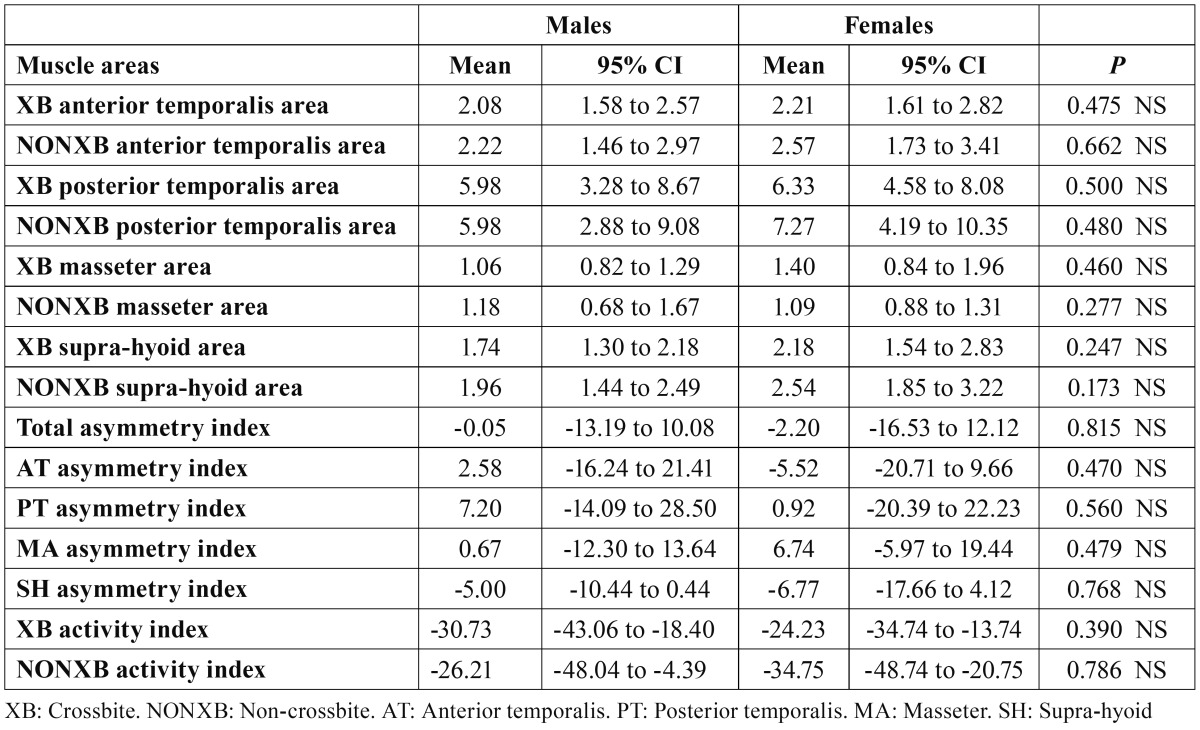
Comparisons between sexes of sEMG muscle areas activity (µV), asymmetry and activity indexes (%) at rest.

**Table 3:**
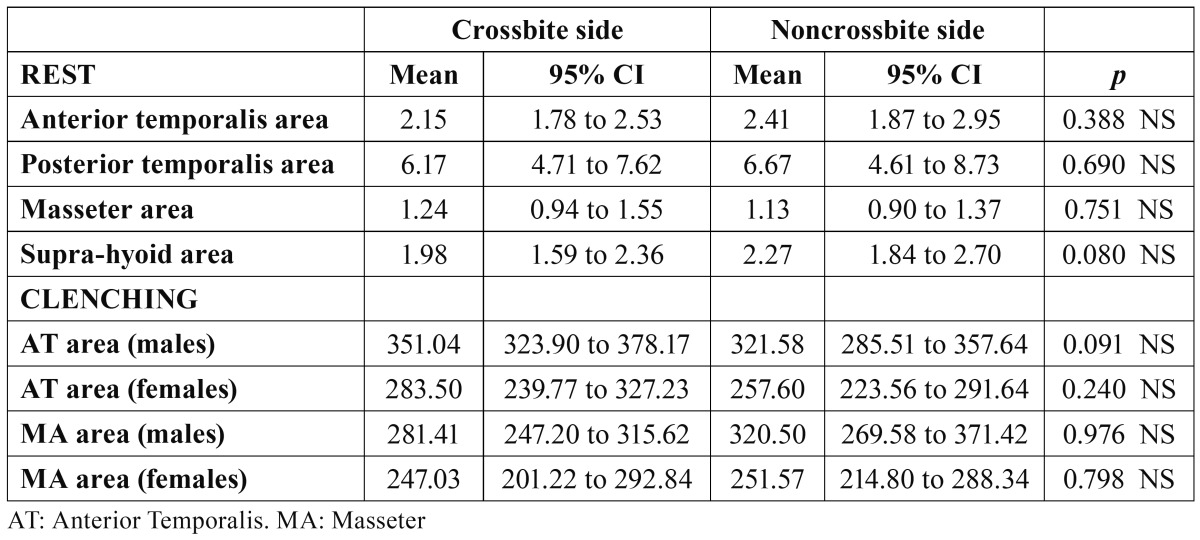
Comparisons between crossbite and noncrossbite side muscle areas at rest (males and females grouped) and during clenching (males and females separately).

No sex differences were found in either asymmetry or activity indexes (T Table 2). Asymmetry indexes showed a symmetric sEMG muscular activity at mandibular rest position. On both XB (males, −30.73; females, −24.23) and NONXB (males, −26.21; fe-males, −34.75) sides, activity indexes showed a predominance of AT over MA activity.

-Clenching

During MVC, AT and MA muscle areas showed statistically significant differences when compared by sex, with higher values in males than in females. Asymmetry indexes, activity indexes and MA/AT ratios did not show significant differences between sexes ( Table 4). In both groups, MA/AT ratios showed a slight predominance of the AT over the MA on the XB side (MA/AT ratio < 1), while on the NONXB side, the contributions of AT and MA were quite similar (MA/AT ratio 1).

**Table 4:**
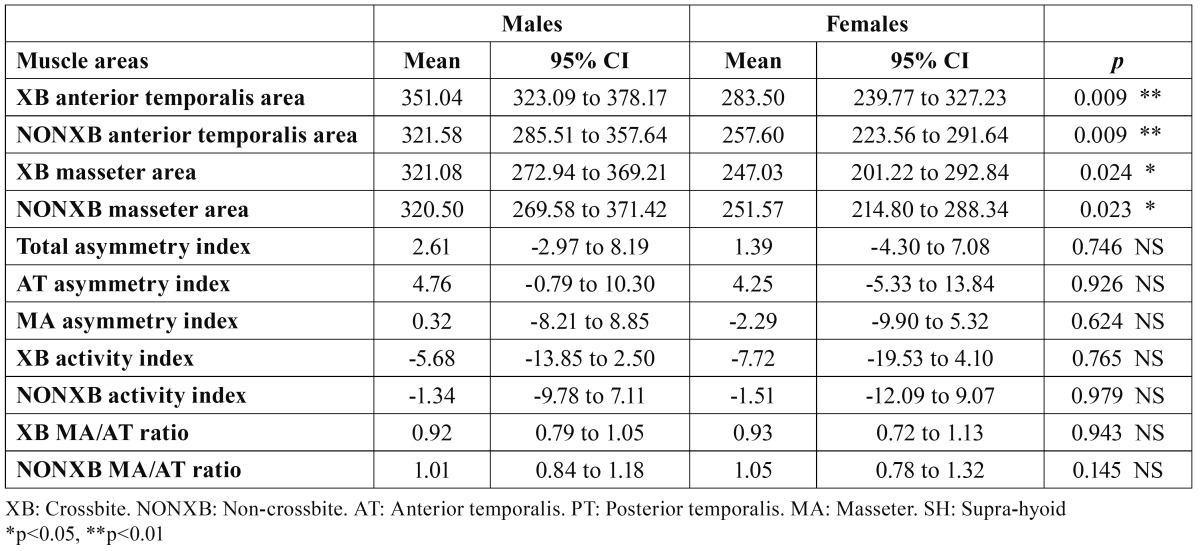
Comparisons between sexes of sEMG muscle areas activity (µV), asymmetry and activity indexes (%) and masseter/anterior temporalis ratios during clenching.

When comparing between sides ( Table 3), no significant differences were found, indicating symmetrical AT and MA activity in both males and females during MVC.

## Discussion

SEMG activity of jaw muscle areas at rest and during MVC in children with UPXB and functional mandibular lateral shift in the early mixed dentition, as well as sex differences, were evaluated. At rest, no differences were found between genders for any muscle areas, but during MVC, significant sex differences were found in the activity of AT and MA in both XB and NONXB sides, with higher sEMG values in males than in females. No asymmetry was found in muscular activity at rest or during MVC in any of the groups.

The sEMG method was chosen because it shows good reproducibility and provides accurate data (22), and it is painless and innocuous, the latter being important factors for studies in children. Nevertheless, the wider intersubject variability found, reflected by the large CIs, should be taken into account in interpreting the results. As noted previously (27), sEMG picks up activity from neighboring muscles. For that reason, we referred to areas instead of individual muscles.

-Rest position

At rest, sEMG values ranged between 1,06 µV (XB MA, males) and 7.27 µV (NONXB PT, females), with no significant differences in any of the eight muscle areas studied, nor in the asymmetry or activity indexes between sexes or sides. Other authors found asymmetry in the activity of the PT (7-9)—even in the AT and MA (10) of children with UPXB—but their samples included older children whose jaw muscles could be more easily affected by the untreated XB, leading to asymmetrical muscular activity between the XB and NONXB sides.

The lack of differences between the sides at rest position found in our study is a remarkable result, as we would have expected asymmetrical activity to accompany the lateral shift. So the presence of UPXB and functional mandibular lateral shift does not seem to affect the symmetric sEMG activity of the PT, AT, MA or SH muscle areas in children in the early mixed dentition stage, at least at this level of mandibular lateral shift (median, 3 mm). Similar results were obtained by Alarcon et al. (27) in children with UPXB but without mandibular shift. It might be necessary extend the time period or investigate a more mature dentition stage (with more occlusal contacts) to elicit such effects as asymmetrical jaw muscle activity at rest position. It might also be clinically and therapeutically useful, in order to establish a suitable baseline, to determine whether there is a moment at which an untreated UPXB with functional shift would generate asymmetrical jaw muscle activity at rest,.

The PT areas showed higher activity (median, XB PT, 6.17 µV; NONXB PT, 6.67 µV) than the other muscles studied and higher than that found in the normo-occlusive control group of Alarcon et al. (median, right PT 3.51 µV; left PT 3.19 µV) (27), as the latter children were the only comparable normo-occlusive group found, including PT. The PT muscles of children with UPXB and functional shift would be expected to exhibit more activity at rest to achieve better stabilization and positioning of the mandible.

We were unable to find any studies dealing with sexual dimorphism in children with UPXB in the early mixed dentition. In studies on the adult normo-occlusive population, no differences between sexes were found in sEMG activity or jaw muscle coordination at rest (22,23,26).

-Clenching

During MVC, maximal sEMG activity, asymmetrical jaw muscles, activity indexes and MA/AT ratios were analyzed and compared between sexes. This test was used to determine to what extend UPXB affects the functional capacity of the jaw closing muscles, because during MVC in maximal intercuspidation, these muscles exert their maximal activity (29).

Significant sex differences were found in sEMG activity of both the XB and NONXB AT (p = 0.01) and MA (p < 0.05), with higher values in males than in females. These results suggest a lower functional capacity of females with UPXB and mandibular lateral shift in the early mixed dentition to develop forces during MVC. These sex differences could involve a different evolution of the malocclusion and different side effects in females, if left untreated, than in males. Moreover, these differences could have some clinical and treatment implications that must be considered and fully investigated in long-term studies.

We found no studies on jaw muscle activity in normo-oclusive children nor in children with UPXB in the early mixed dentition stage comparing differences between sexes. In normal young adults, some studies also found sexual differences, with higher sEMG activity in MA (22) or higher activities in both MA and AT (25) in men than in women.

In healthy adult populations, sexual dimorphism in the muscular system is common, the skeletal muscles of males being capable of producing more force than the same muscles in females. These sex differences have been attributed to differences in muscle mass, fiber-type composition and thickness of masticatory muscles between males and females (20,21,29). Tuxen et al. (18) reported that the greater MA muscle force in healthy young men was associated with their greater diameter and cross-sectional area of type II fibers compared with those of women. They concluded that sex hormones were the main factor in determining the differences in composition and fiber types of MA muscle between males and females. Some other factors have been proposed to explain the differences in fiber-type composition of skeletal muscle, such as genetic factors (19). Future specific studies are recommended to determine whether sex hormone levels present in the early mixed dentition stage explain the differences found between sexes in AT and MA activities.

AT and MA sEMG activities were symmetrical in both males and females, as XB and NONXB muscle area comparisons and asymmetry indexes showed. Similar results were found by Alarcon et al. (27) in a group of children (10–12 y) with UPXB without functional mandibular lateral shift, and by Ingervall and Thilander (8) for AT and MA, in a group of older children (8–12 y) with a lateral forced bite. Nevertheless, other authors found asymmetrical activity in AT (XB AT showed higher activity than the NONXB AT) and MA (NONXB MA showed higher activity than the XB MA) in UPXB in the mixed dentition (10), while others (30) found a higher activity in the XB MA than in the NONXB MA in a group of children with UPXB (7–10 y).

The symmetry found in our subjects might reflect a successful adaptation of the neuromuscular system to the UPXB, at least at this early degree of dental development and growth, as suggested before.

The low activity indexes and the AM/AT ratios close to 1 indicate similar activities of the AT and MA, or even a slight predominance of the AT over MA on the XB side during clenching, in both sexes. These results reflect an imbalance in the activity of the elevator jaw muscles and, therefore, a reduction in the functional capacity of the MAs (mainly on the XB side) in children with UPXB and lateral shift in the early mixed dentition, as the most physiological explanation for the predominance of the MA over AT during clenching (22).

## Conclusions

SEMG analysis of the jaw muscles in children with UPXB and functional mandibular lateral shift in the early mixed dentition showed no sexual dimorphism at rest, but in MVC, significant sex differences were found in the activity of both XB and NONXB TA and MA muscle areas, with higher values in males than in females. Muscular activity was symmetrical at rest and during MVC in both sexes. Sexual dimorphism should be considered in diagnosing and treating children with UPXB and lateral shift in the early mixed dentition. Long-term studies with larger samples are needed to investigate the potential consequences of sex differences during MVC if the malocclusion remains untreated and to determine the implications for treatment.
